# Brain organoids: A new tool for modelling of neurodevelopmental disorders

**DOI:** 10.1111/jcmm.18560

**Published:** 2024-09-11

**Authors:** Yirizhati Aili, Nuersimanguli Maimaitiming, Zengliang Wang, Yongxin Wang

**Affiliations:** ^1^ Department of Neurosurgery The First Affiliated Hospital of Xinjiang Medical University Xinjiang People's Republic of China; ^2^ Key Laboratory of Precision Diagnosis and Clinical Transformation of Nervous System Tumors Xinjiang Medical University Xinjiang People's Republic of China; ^3^ The Cancer Institute and Hospital Chinese Academy of Medical Sciences Beijing People's Republic of China

**Keywords:** brain organoids, induced pluripotent stem cells, neurodevelopmental diseases, preclinical models

## Abstract

Neurodevelopmental disorders are mostly studied using mice as models. However, the mouse brain lacks similar cell types and structures as those of the human brain. In recent years, emergence of three‐dimensional brain organoids derived from human embryonic stem cells or induced pluripotent stem cells allows for controlled monitoring and evaluation of early neurodevelopmental processes and has opened a window for studying various aspects of human brain development. However, such organoids lack original anatomical structure of the brain during maturation, and neurodevelopmental maturation processes that rely on unique cellular interactions and neural network connections are limited. Consequently, organoids are difficult to be used extensively and effectively while modelling later stages of human brain development and disease progression. To address this problem, several methods and technologies have emerged that aim to enhance the sophisticated regulation of brain organoids developmental processes through bioengineering approaches, which may alleviate some of the current limitations. This review discusses recent advances and application areas of human brain organoid culture methods, aiming to generalize optimization strategies for organoid systems, improve the ability to mimic human brain development, and enhance the application value of organoids.

## 1 INTRODUCTION

The study of embryonic development and late organ maturation processes has always been a major challenge due to the limitations of obtaining and culturing human embryonic tissues.^1^ Hence, establishment of in vitro systems that can mimic the complex processes of human organogenesis will go a long way towards solving this difficulty.^2^ Three‐dimensional (3D) aggregates formed by human induced pluripotent stem cells (iPSCs) can generate an astonishing level of self‐organization, which can then evolve through cell–cell interactions and differentiate to form highly organized organ‐like structures.^3^ Pioneering work in the Yoshiki Sasai laboratory^4^ has demonstrated autonomous emergence of highly ordered neural structures from 3D aggregates formed from embryonic stem cells (ESCs). Based on this technology, a number of strategies to develop organoids based on specific regions of the brain have emerged in the last decade; however, the extent to which organoids can mimic actual neural development determines the potential application of organoids as models for neurological disease research.^5^ Therefore, a systematic understanding of organoids as a multicellular system and mastery of methods to regulate cellular self‐organization will enable us to make full use of the potential of applications of organoids and further optimize organoid systems to more closely resemble in vivo developmental processes, thereby overcoming the limitations of organoids.

Neurodevelopmental disorders (NDD) are disorders of the nervous system caused by genetic or acquired causes that result in abnormal development of the nervous system, which in turn gives rise to brain dysfunctions, including mental retardation, autism spectrum disorders, attention deficit hyperactivity disorder and other disorders.^6^ Therefore, resolving the pathogenic mechanisms of neurodevelopmental diseases is one of the key topics that is being investigated in the field of neurobiology. Nevertheless, due to limitations of ethics and hurdles in obtaining human and non‐human primate brain tissues, the few brain tissues that can be obtained only reflect the end stage of a disease but cannot actually resolve the underlying mechanisms of disease progression.^7^ As a result, understanding of developmental diseases of the human brain has been mainly derived from studies in rodents.^8^, ^9^ Therefore, a new model that can better recapitulate the characteristics of the human brain is urgently needed for a more in‐depth understanding of development, functions and disease progression of the brain.

The progression in stem cell research, notably the innovation of iPSCs, has provided ideal human cell models for elucidating the pathogenesis of neurodevelopmental disorders.^10^ Disease models using somatic cells of patients that carry specific disease genes can be reprogrammed to form iPSCs that subsequently differentiate into various types of neural cells. Such models have been widely to study various neurological diseases.^11^ However, neurological disease phenotypes, which include structural brain abnormalities such as brain size and synaptic activity, are known to be heterogeneous. Conventional two‐dimensional (2D) neural cultures provide limited insights into this complexity;^12^ hence, 3D brain organoids culture protocols have been pioneered by improving the 2D neural culture methods, thus opening new horizons for the study of neurodevelopmental diseases.^13^

Additionally, brain organoids offer an advantageous platform for screening of drugs in the central nervous system, circumventing the limitations of traditional 2D neural cultures.^14^ Notably, brain organoids possess characteristics such as tissue organization and cell diversity that closely resemble natural brain development. Moreover, insights into the integration of different brain regions can be gained through in vitro modelling using brain organoids.^15^ The use of brain organoids combined with emerging technologies related to gene editing, fused organoids, organ microarrays and single‐cell RNA sequencing can break through the bottleneck of using traditional models and provide valuable information for disease modelling, drug discovery, development, precision medicine and regenerative medicine at the organ level.^16^ In this article, we review history of the establishment and development of in vitro models of neurological diseases, present the latest research data on brain organoids for exploring neurodevelopment and disease pathogenesis, and provide an overview on the application of cutting‐edge technologies related to brain organoids.

## 2 PROGRESS IN RESEARCH ON BRAIN ORGANOIDS

Brain organoids, which are self‐organized 3D cellular structures generated in vitro, can be used to study neurodevelopment and neural functions. iPSCs can differentiate into specific brain regions such as the dorsal or ventral forebrain in response to developmental signalling factors or can give rise to a variety of neural and non‐neural cell lineages through undirected differentiation.^17^ 3D organoid culture technology has greatly contributed to the development of a new generation of novel studies in developmental neurobiology.^18^ For several years, scientists have relied on models of animal brains that exhibit significant differences in structure, function and development compared to those of the human brain. In recent years, organoids of the brain have been shown to mimic many of the typical and unique properties of neurogenesis in vivo, especially cortical developmental processes, thus making them an excellent and novel approach to study endogenous features of the human brain.^19^, ^20^

### 2.1 Importance of brain organoids

Brain organoids are 3D models of simulated human brains cultured in vitro that incorporate many aspects of human brain tissue, including cellular structure of early brain development, cellular variability, cell–cell and cell‐substrate interactions, as well as characteristics of several cell types, which are found to be lacking in 2D monolayer cultures. Unlike 2D cultures, 3D organoid models can reproduce the 3D anatomy of tissues; such in vitro models can closely resemble in vivo tissue structures. Organoid systems have the potential to generate a wide range of cellular diversity, making it possible to study cell–cell interactions (neurons and glial cells) during early developmental processes.^21^ Yakoub et al.^22^ demonstrated that brain organoids obtained under optimal differentiation conditions exhibit mature fully functional neurons and astrocytes, as confirmed by immunohistological analysis, gene expression studies and electrophysiological analysis. In addition, by inducing differentiation to generate different regions of the brain and their cellular components, brain organoids can further mimic connecting networks between different parts of the brain.^23^ This study suggests that organoid models of the brain can encapsulate many features of the developing brain, such as radial organization of periventricular cell types found in the early neural tube.^24^ Neural stem cells derived from iPSCs initially generate deep neurons, followed by upper neurons, and it has been shown that the sequence of cell production is similar to the process of neural development in the human body. This property allows brain organoids to serve as one of the models for elucidating human brain development and disease.^25^

**FIGURE 1:**
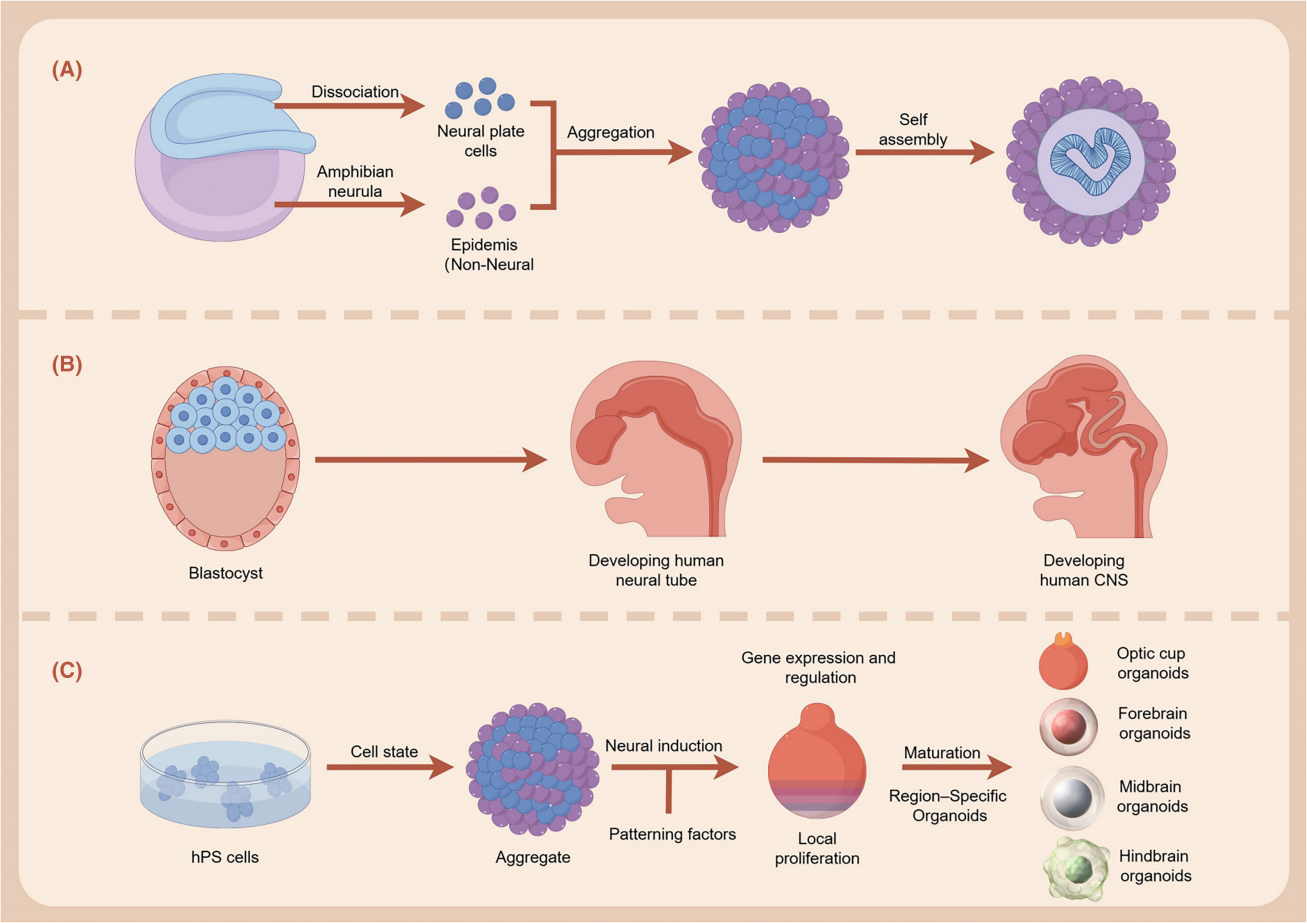
Human brain organoids recapitulate key processes in human brain development: (A)The ectoderm develops further around embryonic day 20 to form the neural plate in the centre, the surface ectoderm on either side, and the neural plate border in between. The neural plate bends, folds, thickens and closes further to form the neural tube. (B)The head end of the neural tube forms a Blastocyst, which eventually forms the parts of the brain. Each part of the brain vesicle grows at a different rate, forming three enlarged parts: The forebrain, the midbrain, and the rhombencephalon. Formation of a more complete central nervous system by the sixth week of development. (C)Brain organoids are well able to simulate in vivo processes such as neurogenesis, neuronal migration, cortical stratification and neural loop establishment.

Brain organoids derived from human iPSCs are more compatible with the genetic makeup and structural features of the human brain than animal models. For certain neurodevelopmental diseases associated with large‐scale structural rearrangements and multiple genes, it is not feasible to establish a compliant animal model; however, organoids can be reprogrammed into iPSCs using somatic cells from such patients, which can then be induced to differentiate into brain organoids and establish disease models.^26^, ^27^ Several studies have used brain organoids as disease models to study congenital brain malformations caused by genetic defects. Detection of primitive microcircuits and spontaneous neural activities in brain organoids, as well as further promotion of functional maturation of organoids can simulate diseases related to brain development.^28^ In addition, brain organoids are more readily available and can be generated in greater numbers than human brain tissues, which are difficult to obtain (especially embryonic tissues).^29^ Finally, compared to using other animal models, using organoids allows for the study of unique human aspects of brain formation and evolution. Brain organoids generated from human iPSCs are comparable to human cell‐derived organoids, which can uncover unique human developmental features.^30^ In summary, brain organoids can mimic developing human brain tissue and can be employed to study development, evolution and neurological diseases of the brain (Figure 1).

### 2.2 Mechanism of differentiation of human brain organoids

Human brain organoids are differentiated from human induced pluripotent stem cells (hiPSCs) and human embryonic stem cells (hESCs) under 3D culture conditions, relying on the spontaneous morphogenesis of cell aggregates.^31^, ^32^ Two methods are used to differentiate hPSCs into organoids, namely, non‐guided (non‐directed) and guided (directed) differentiation methods (Table 1).

**TABLE 1:** Diverse protocols for brain organoid generation.

Main type	Differentiation strategy	Organoid identity	Culture system	Characteristic	References
Whole brain organoids	Unguided	Whole brain Whole brain Forebrain Forebrain	Suspension culture, suspension rotary bioreactor Suspension culture, suspension rotary bioreactor Microfilaments, suspension culture, Horizontal shaker, air liquid interface Microfilaments, horizontal shaker	Apicobasalcell polarity, Internuclear ophthalmoplegia, Neural stem cell division and neuronal migration patterns, The Outer subventricular zone (OSVZ) is enlarged Diverse cells, including cells such as the cortex and retina, neuronal maturation, spontaneous neuronal network formation Neurons are projected long distances, with functional output of the cortex and callosum tracts The cortex is well developed, the cortical plate is polarized, and the radial tissues and neurons of the cortex migrate	Benito [33] Lancaster [34] Quadrato [35] Giandomenico [23]
Region‐specific organoids	Guided	Cortex Cortex Cortex Cortex Cortex Cortex Optic cup Ventral forebrain Hippocampus Choroid plexus Medial ganglioniceminence (MGE) Thalamus Hypothalamus Pituitary gland Hypothalamus Midbrain Posterior brain (cerebellum) Brainstem Neuromuscular	Floating culture in 40% O_2_/5% CO_2_ Suspension Suspension Suspension, rotating bioreactor Suspension, Rotating horizontal shaking table Suspension, rotating bioreactor, rotating horizontal shaking table and slice suspension Suspension Suspension, 40% O_2_/5% CO_2_ Suspension Suspension, 40% O_2_/5% CO_2_ Suspension, rotating horizontal shaking table Suspension, rotating horizontal shaking table Suspension, rotating horizontal shaking table Suspension, rotating bioreactor Suspension, 40% O_2_ Suspension, rotating shaker Suspension, rotating bioreactor Suspension Suspension, rotating shaker Suspension, rotating horizontal shaking table	The formation of axial polarity and the generation of a hemispherical structure are observed in self‐organizing cortical tissue. The neuroepithelium exhibits a multi‐layered composition, comprising three distinct neuronal regions (inferior lamina, cortical lamina and Cajal Retzius cell regions) and three progenitor cell regions (ventricular region, subventricular region and intermediate region) Form cortical neurally in a spatially and temporally controlled pattern Organs consist of deep and surface cortical neurons exhibiting electrophysiological activity, enveloped by astrocytes and establishing functional synapses Reproducing key feature of human cortical development, such as the presence of progenitor cell regions, neurogenesis, gene expression and notably the distinctive outer radiating glial cells specific to humans Outer radial glial cells, which encompass astrocytes, manifest a radial morphology and are present in both the superficial and deep layers of the cortex The forebrain organs demonstrate the presence of several progenitor cell regions, which collectively form an expanded cortical plate responsible for generating various neuronal subtypes across six cortical layers Produces retinal pigment epithelium, neuroretina, consisting of six. The cells in the sensory layer of the retina appear to be valgus folded phenomenon Expression of the ventral forebrain markers NKX 2‐1, DLX 2 and GSX 2 The generation of hippocampal granule neurons and pyramidal neurons culminates in the establishment of a cohesive and operational network The formation of a cubic epithelium results in the production of ‘cysts’ or compartments that contain secretions resembling cerebrospinal fluid, thereby establishing a highly interconnected barrier During the process of maturation, MGE type organs exhibit the presence of intermediate neurons, including GABAergic and SST+ neurons. Additionally, the migration of progenitor cells is observed within OSVZ‐like structures The thalamic organs exhibit a high level of expression for OTX2, GBX2 and DBX1, mirroring the expression patterns observed in thalamic neurons in vivo Following a period of 40 days, organoids exhibited the emergence of peptidogenic neuronal markers, accompanied by the expression of OTP, a homeobox protein that plays a critical role in the differentiation of hypothalamic lineages Simultaneous development of hypothalamic and anterior pituitary neurons results in the maturation of hypothalamic tissue and subsequent secretion of adrenocorticotropic hormone Markers such as OTP, DLX, TBX3 and POMC are expressed during the developmental process of the hypothalamic arcuate nucleus, encompassing diverse neuronal populations. Functional dopaminergic neurons are observed in the intermediate and advanced stages of brain‐like structures Dopaminergic neurons with functional properties are observed in the intermediate and advanced developmental stages of midbrain‐like structures. Produce cerebellar plate epithelium with diamond like structure, containing electrophysiological active Purkinje cells This composition encompasses progenitor cells originating from the midbrain and hindbrain, as well as noradrenergic and cholinergic neurons, dopaminergic neurons, and neural crest lineage cells Human neural mesodermal progenitor cells derived from pluripotent stem cells have the capacity to generate human neuromuscular organs in a 3D culture system, resulting in the formation of functional neuromuscular connections	Kadoshima [36] Eiraku [37] Paşca [38] Qian [39] Jovanovic [40] Qian [41] Nakano [42] Birey [43] Sakaguchi [44] Pellegrini [45] Xiang [46] Xiang [47] Shao [48] Kasai [49] Huang [50] Jo [51] Muguruma [52] Eura [53] Faustino [54]

#### 2.2.1 Non‐guided methods for organoid generation

Non‐guided differentiation primarily exploits the spontaneous morphogenesis and intrinsic signalling potential of human pluripotent stem cell aggregates to generate brain organoids that contain features of multiple cell lineages.^55^ In non‐guided differentiation, hPSCs are grown in a stromal gel suspension (e.g., Matrigel), with the advantage that they can develop into a variety of cell lineages, including those of the dorsal forebrain, ventral forebrain, midbrain, hindbrain, hippocampus, retina, choroid plexus and even non‐neural lineages.^34^, ^56^

Single‐cell transcriptome analysis has shown that brain organoids contain dorsal and ventral forebrain cells, cells from other brain regions (retina, hindbrain and midbrain), choroid plexus and mesodermal cells. However, spontaneous differentiation is highly stochastic; hence, the proportion and arrangement of cell lineages in each organoid is unpredictable.^35^, ^57^ Although coexistence of various cell lineages can be used to study interactions between different brain regions, high variability between batches and cell lineages makes systematic and quantitative studies difficult. Additionally, early existing conditions can have an impact on the development of brain organoids, which may lead to non‐physiological cellular interactions if certain cell populations are recognized and eliminated during development. The variability observed in non‐guided brain organoids may be related to inconsistent neural induction, and currently small molecules^58^ or fibrous microfilaments^34^ are employed to limit the fate of cells and reduce their variability.

Furthermore, non‐guided organoids contain various types of neuronal and glial cells from multiple brain regions and have been shown to contain a wide range of cell types from non‐epidermal lineages. This type of cell culture technique compromises the reproducibility of organoid cultures and has limited applications in disease modelling and drug screening.^59^ Therefore, targeted induction of organoid culture modalities related to specific regions of the cerebral cortex has emerged as a widely used alternative in this field and generates highly reproducible region‐specific brain organoids.

#### 2.2.2 Guided methods for organoid generation

In guided differentiation methods to generate organoids, aggregated hPSCs are assigned an ectodermal fate and subsequently develop into region‐specific organoids or organospheres.^37^, ^60^ Directed differentiation is based on the principle of neural differentiation, which uses small molecules and growth factors to form cells and structures that represent specific brain regions. Typically, neural profiling is promoted by inhibition of the BMP/TGF‐β signalling pathway, and the subsequent use of associated growth factors (e.g. WNT3A, SHH, BMP7, FGF8, FGF2 and insulin) allows for further directed differentiation of brain organoids related to specific brain regions. The earliest study in this field was by Eiraku et al.,^37^ who cultured ESCs in U‐or V‐shaped bottom wells using serum‐free embryoid bodies with quick reaggregation (SFEBq) and demonstrated that ESCs were able to self‐organize to form parietally polarized cortical tissue. Additionally, the cell cycle could be ended at regular intervals according to the simulated order of appearance of different cortical neurons, thereby selectively generating specific neurons.

Subsequently, Pasca et al.^38^ proposed a method for culturing the 3D dorsal forebrain with neural differentiation using a suspension culture of hiPSC cells, without the need for an extracellular matrix or bioreactor culture conditions. These globular cell clusters can grow up to 4 mm in diameter, contain deep and superficial cortical neurons as well as non‐reactive astrocytes, and mature after approximately 9 to 10 months, thus resembling a mature brain structure at the postnatal stage. Similarly, Qian et al.^39^ developed a miniature rotating bioreactor to generate forebrain‐specific organoids from hPSCs that share key features of human cortical development, including progenitor region organization, neurogenesis, gene expression and a glial cell layer that is unique to humans. Based on this methodology, the researchers established a model based on exposure to Zika virus (ZIKV) and derived midbrain or hypothalamic organoids. Currently, many specific brain region structures, such as the dorsal forebrain cortex,^40^, ^41^ ventral forebrain,^43^ hippocampus,^44^ thalamus,^47^ hypothalamus,^48^ pituitary gland,^49^, ^50^ choroid plexus,^45^ midbrain^51^ and cerebellum^52^ are created employing the directed differentiation technique.

Region‐specific brain analogs can be used in brain assembly studies, wherein such analogs are fused together to study interneuron migration, neuronal projections and oligodendrocyte generation.^61^ Glial cells play an important role in the regulation and support of the nervous system. Although astrocytes and oligodendrocyte progenitors have been observed to grow in cortical organoids after long‐term static culture, development of mature oligodendrocytes has not been observed.^33^ The researchers established region‐specific brain organoids for oligodendrocytes, astrocytes and neurons, which can be used to study the system of oligodendrocyte development, myelin formation and interactions with other cell types.^21^ Microglia are immune cells of the nervous system that regulate its health by responding to inflammation, phagocytosing infectious microbes and pruning redundant synapses. Consequently, region‐specific brain organoids with microglial cells can be used to study microglia migration and responses to damage in a 3D environment.^62^

Compared to the non‐guided differentiation approach, the guided differentiation approach to obtain specific brain organoids has been shown to vary less between batches and cell lines, making the experiments more reproducible, quantitative analyses easier and more reliable, and suitable for a wide range of neurological disease modelling and drug screening.

### 2.3 Human brain organoid culture methods

**FIGURE 2:**
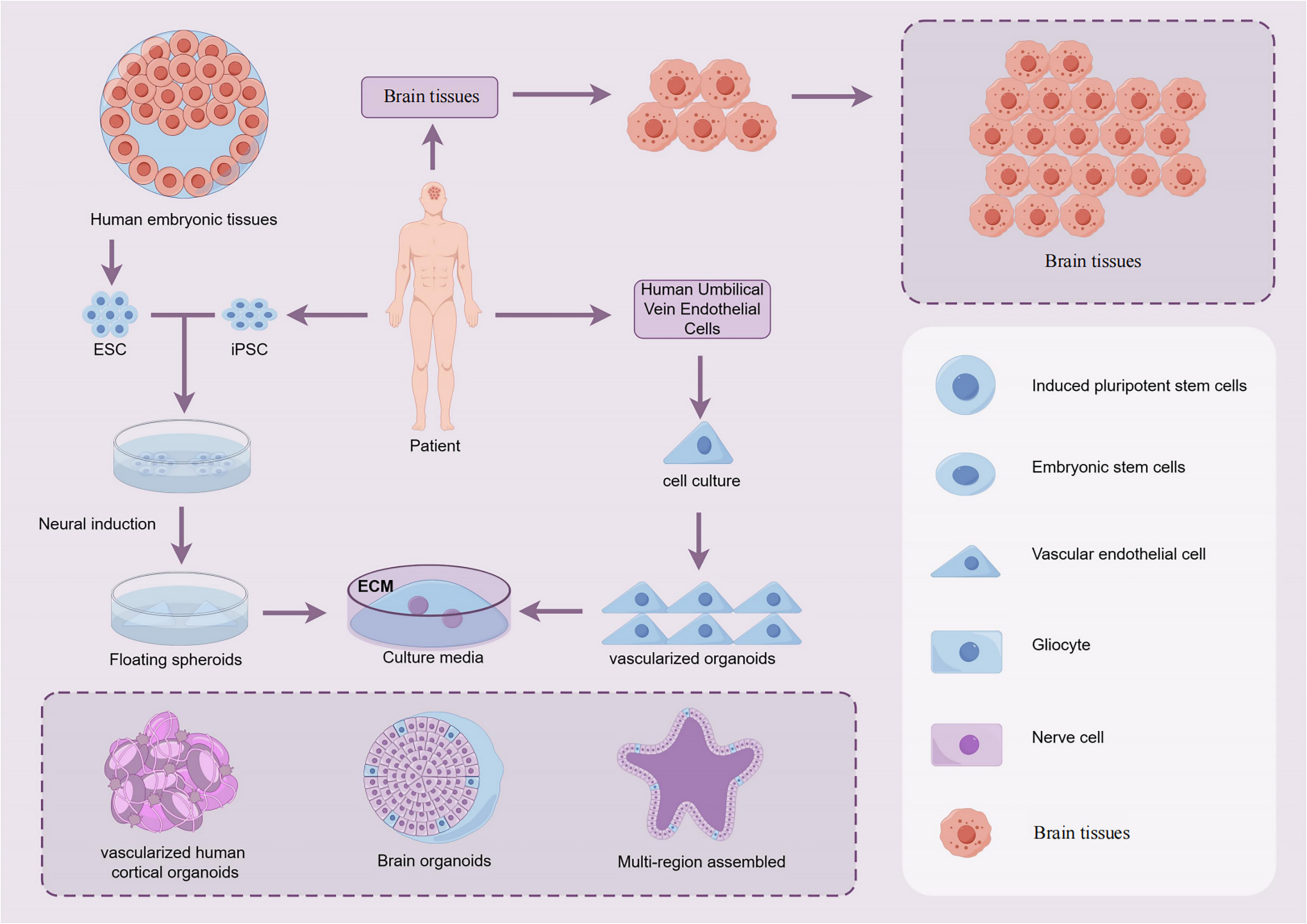
Diferent types of human stem cell/organoid‐derived tumour models: IPSCs are derived from patient donor tissue or healthy family control subjects. These iPSCs are then used to generate organoids specific to different brain regions by mimicking the natural development process of the human brain with appropriate morphogens. Comparative analysis of patient and control organoids can reveal pathological phenotypes and mechanisms at molecular, cellular, structural, and functional levels. Utilizing insights from these studies, brain region‐specific organoids and human vascularized brain organoids can be employed for detecting drug responses and facilitating large‐scale drug screening. Additionally, human cancer tissues, which comprise cancer cells, adult stem cells, and pluripotent stem cells, are sometimes initially separated into small fragments, cell clusters, or single cells using mechanical and chemical methods. These are then cultured in 3D hydrogels containing extracellular matrix components to create patient‐derived brain organoids.

The advancement of brain organoid development has been significantly enhanced by the integration of diverse engineering technologies, including static culture, microfluidic devices and vascular‐like system. These innovative approaches have not only minimized structural heterogeneity but also significantly improved the development and maturation of neuronal tissues (Figure 2).

#### 2.3.1 Long‐term static culture

In a static culture system, cells are cultured under physiological fluid dynamics in the absence of nutrients or waste. One approach for generating brain organoids is to use neural‐inducing molecules to promote cell aggregation and self‐organization. In this method, hPSCs are placed in low adsorption cell culture plates, which allow the cells to self‐assemble into spherical shapes, grow continuously and display layers of cortical neurons and non‐reactive astrocytes.^43^ Another method of enabling cell aggregation is the use of U‐ or V‐bottom perforated plates, in which suspended cells are collected at the bottom and self‐organize into 3D spheres due to the curved shape and low adsorption of the holes.^63^ Lancaster et al.^55^ embedded brain organoids collected in U‐bottom plates in matrigel and transferred them to a rotating bioreactor to improve oxygen diffusion and nutrient distribution. Although brain organoids develop rapidly and show polarized features of neural precursor cell regions, static culture methods are prone to display necrosis within the core of the brain organoids due to inadequate perfusion and fluid flow caused by insufficient nutrients and exchange of gases and wastes.^64^

#### 2.3.2 Human organotypic brain slice cultures

Organotypic brain slice culture techniques can greatly improve the oxygenation capacity of brain organoids and reduce the formation of hypoxic cores. Using gas–liquid interface culture technique to culture the organoids, mature organoids are embedded in agarose and sliced into 300 μm slices, which can be cultured for several months in a specific medium.^23^ Compared to static culture, this culture method can improve neuron survival and promote axon growth, and the resulting thick axon bundles exhibit multiple morphologies. In addition, subcortical projection bundles can innervate mouse spinal cord outgrowths to cause contraction of neighbouring muscles. Qian et al.^41^ also developed a forebrain organoid sectioning system, in which, after ventricular zone (VZ) and radial glial cell scaffolds are established, the forebrain organoids are embedded in agarose, sliced into 500 μm thick sections using a vibrating slicer, and placed on an orbital shaker to prepare a suspension culture. The disc‐shaped organoid slices received oxygen and nutrients by diffusion through the exposed top and bottom surfaces, and the slices grew horizontally while maintaining the structure of the cortical tissue. Compared to cultured static forebrain organoids, sections cultured to 140 days showed a significant reduction in the area of necrosis within the core and on the external layer of the organoids, and displayed continued neurogenesis and cortical plate expansion, with the cortical plates exhibiting distinct deep and shallow cortical layers.

Organotypic brain slice culture techniques can overcome the effects of diffusion barriers on the growth of organoids and address the problem of hypoxia within the tissue, while improving nutrient delivery and maintaining tissue expansion.^65^ However, the process of cutting the tissues mechanically is prone to contamination, and the slicing technique can lead to tissue flattening, disrupting the structure of the VZ and subventricular zone (SVZ).

#### 2.3.3 Microfluidic co‐culture systems

In recent years, microfluidic co‐culture systems have been applied to brain organoid cultures and can be performed in vitro while having the advantage of better mimicking of in vivo environment.^66^ Currently, a typical method of preparing microfluidic devices is to etch patterns onto silicon or polymer moulds made of biocompatible and flexible materials, pour polydimethylsiloxane (PDMS) into the moulds, remove them, and bond them to form microfluidic channels.

Microfluidic co‐culture systems can be divided into static and dynamic culture systems. The purpose of the static culture is to achieve a one‐stop culture of brain organoids and to simplify the operation process. The microcolumn array can generate brain organoids from hiPSCs in situ, and single cells are effectively aggregated into embryoid bodies (EB) under the action of the microcolumns, followed by neural induction and differentiation, which achieves in situ assembly of brain organoids. This method simplifies the operational steps, reduces cell contamination during experiments, and improves efficiency of brain organoid generation. A study reported the use of a six‐well plate‐based porous “filter” PDMS chip having 169 cylindrical holes of 2 mm diameter each and a PTFE‐modified metal mesh fixed at the bottom of the microwells to generate small organoids.^67^ A specific number of hESCs were implanted into each hole, and the hydrophobic mesh allowed the cells to spontaneously aggregate into EBs, which then continued to undergo neural induction, expansion and maturation in the holes. The entire process requires only one culture vessel, which prevents EBs from being lost or damaged during transfer and avoids potential contamination risks.^68^ The limitation of the physical size of the wells was utilized to produce 169 brain organoids of less than 2 mm per batch, thereby reducing the size variation of organoids generated within the same batch and preventing the formation of uneven necrosis.

The human brain is a highly dynamic organ with neural tissue embedded in extracellular matrix; in a dynamic culture system, constant infusion of nutrients and removal of wastes increases intracellular exchange of nutrients and wastes and reduces organoid necrosis.^69^ A study reported that a pumped microfluidic system can be used to culture midbrain organoids by placing the organoids in six‐well chips, wherein each six‐well chip is connected to a reservoir bottle containing 22 mL of medium; this loop is connected to a peristaltic pump to ensure supply of medium through a continuous laminar flow. This culture condition resulted in higher oxygen levels in the core region of the midbrain‐like organoids, a much smaller necrotic core than that observed in conventional culture, and increased differentiation of neural stem cells to dopaminergic neurons.^70^ While pump‐based dynamic culture systems can improve the culture of brain organoids, the use of pumps increase the size of the system and make the culture more complex; therefore, researchers have used a number of techniques to simplify the culture system while maintaining a constant flow of fluid within the chip.^71^ One method involves the use of hydrostatic pressure, which creates a pressure difference by varying the height of the gas–liquid interface between the inlet/outlet reservoir liquids and the atmosphere.

This device produces a flow rate comparable to the interstitial flow rate, which promotes differentiation of neural progenitor cells and extension of neural synapses.^72^ Hydrostatic pressure can also generate low flow rates through the microchannels present between the chambers, promoting axonal extension and synapse formation. Although creating hydrostatic pressure is a simple process that is used in microfluidic devices, the pressure exerted within the microchannels lacks dynamic control. The flow of fluid from one reservoir to another over time results in a nonlinear decrease in pressure, with the flow in the microchannels eventually ceasing when the level within the reservoir reaches equilibrium. Cho et al.^67^ developed a three‐layer, five‐chamber chip with five chambers connected by microfluidic channels (two of which were used for brain organ culture and three for medium storage), in which the fluid flow was driven by water purification pressure generated by an up‐and‐down oscillating shaker, thereby eliminating the need for external piping and drive pumps. The chip‐cultured brain organoids showed increased cell proliferation and reduced cell death. The small size of the pumpless microfluidic culture platform enables more precise fluid control, effective fluid exchange and easy high‐throughput culture.

This type of microfluidic co‐culture system can improve the culture conditions of organoids. On the one hand, it can achieve one‐stop culture of organoids; on the other hand, it can improve the oxygen supply and material exchange in the central region of brain organoids through continuous flow of fluids and reduce death of the central region, which greatly improves reproducibility and consistency of the culture of brain organoids. Through further design and integration, the microfluidic system can achieve high‐throughput culture of brain organoids, which can be applied for drug screening, drug toxicity assessment and in other fields.

#### 2.3.4 Brain organoids with a functional vascular‐like system

The human vascular and nervous systems develop in parallel.^73^ However, current methods of generating brain organoids lack a vascular system, resulting in inadequate delivery of nutrients and oxygen, thus limiting the size and maturation of brain organoids. Although orbital shakers or other devices can be used to agitate brain organoids, they can improve the supply of oxygen and nutrients only to the surface of the brain organoids.^55^ Although the organotypic brain slice culture method can provide oxygen and nutrients to the deep region, the specific 3D structure of brain organoids is destroyed because it is cut several times. Therefore, vascularization of brain organoids is a very important area of research, which is mainly achieved by two methods: in vitro and in vivo cultures.

One approach that can be used for in vitro culture is to co‐culture organoids with vascular cells. Brain organoids and endothelial cells (ECs) were induced using iPSCs from the same source, and subsequently the 34‐day organoids were embedded in a matrix gel containing ECs; thus vascularization of the organoids was successfully achieved.^74^ In another study, human brain organoids were co‐cultured with human umbilical vein endothelial cells (HUVECs); it was observed that in the brain organoids cultured for more than 200 days, vascularization first occurred in the area occupied by neural stem cells and neural progenitor cells. This process is similar to the development of the human cerebral vascular system in vivo.^75^

Reduction in H1F1 and Caspase3 positive cells demonstrated that the vascularization using HUVECs improved the hypoxic state of human brain organoids, which were larger in size, suggesting that vascularization promoted neural progenitor cell proliferation and neuronal survival. In another study, single‐cell RNA sequencing and electrophysiological analyses revealed that vascularized organoids had a higher number of spontaneously active neurons than non‐vascularized organoids and that vascularization accelerated neurogenesis and neuronal maturation in human brain organoids.^76^

Another approach is to combine gene editing technology to create vascularized brain organoids. Human embryonic stem cells were infected with lentivirus to induce the generation of human brain organoids, which contained VZ‐like zones, SVZ‐like zones and cortical tissues, in addition to the usual cultured tissues.^77^ Cakir et al.^77^ found that overexpression of ETS variant transcription factor 2(ETV2) in the organoids induced the formation of ECs, which led to the formation of vascular‐like structures, promoted oxygen diffusion, reduced cell death and facilitated enlargement of brain organoids and neuronal maturation.^77^ In addition, vascularized brain organoids are reported to exhibit several blood–brain barrier features, such as increased astrocyte and pericyte protein expression and transendothelial electrical resistance, which can be used as a model of the blood–brain barrier (BBB).^78^

These vascular‐like brain organoids, although not actually supplied with blood flow, improve oxygen and nutrient diffusion, reduce formation of a hypoxic core and promote development of brain tissues.

In vivo culture is a method of transplanting brain organoids into organisms to establish circulatory vasculature, wherein blood circulation delivers oxygen and nutrients to the organoids on a continuous basis. When 40 to 50 days human brain organoids were transplanted into the postsplenic cortex of immunodeficient mice, approximately 92 percent of the transplanted organs survived for more than 180 days.^79^ In another study conducted in mice, blood vessels significantly infiltrated human brain organoids within 14 days of transplantation, with 85.4% of transplanted‐like organs successfully vascularized, and those that failed to vascularize did not survive, suggesting that transplanted‐like organoids are dependent on blood circulation to deliver oxygen and nutrients to support survival.^80^

Vascularized brain organoids have fewer apoptotic cells, larger volumes and greater numbers of mature neurons than non‐vascularized brain organoids. Pre‐vascularized brain organoids were induced using ESCs that overexpressed ETV2 and transplanted subcutaneously into the hind limbs of immunodeficient mice. Magnetic resonance imaging analysis showed that the pre‐vascularized organoids survived 30 days after transplantation, whereas those that were not pre‐vascularized were undetectable after transplantation. Perfusion with fluorescein isothiocyanate(FITC)‐dextrose demonstrated vascular infiltration in mice, and functional perfusion was established only in pre‐vascularized organs. When brain organoids were co‐cultured with HUVECs for 60 days and then implanted into the somatosensory (S1) cortex of NOD‐SCID mice, pre‐vascularization promoted angiogenesis and reduced apoptosis than in untreated organoids.^77^ Immunohistochemical analysis showed that the capillaries of the brain organoids contained both HUVEC and mouse‐derived endothelial cells, indicating vascular connectivity and functional perfusion between the brain organoids and the host mice. These results suggest that the in vivo transplantation process was able to provide blood circulation to the brain organoids, thus facilitating long‐term survival of the organoids.

There are many differences between human and rodent brains, such as the number of nerve cells, biophysical properties, neurogenesis, BBB permeability, angiogenesis, etc. Animal models cannot fully represent the human brain nor can they directly reproduce the cerebral ischemia model in the human body; therefore, transplantation of brain organoids into the body to establish blood circulation is of great significance for the establishment of ischemic stroke models.^81^ This can be achieved by transplanting brain organoids into immunodeficient mice and connecting them to blood vessels, thereby simulating an ischemic stroke to analyse the role of haematopoietic cells.^81^ In addition to delivering oxygen and nutrients, researchers have found that haematopoietic stem cells play an important role in ischemia, acute inflammation, clearance of damaged tissue and functional recovery after ischemia.^82^ However, vascularized organoids transplanted in immunodeficient mice are perfused by mouse blood, which has a different composition than that of humans. It has been reported that some severely immunodeficient mice can be “humanized” using human haematopoietic stem cell transplantation.^83^ Transplantation of human haematopoietic stem cells into mice after transplanting human brain organoids into immunodeficient mice enable the mice to have both human brain organoids and human blood cells. Therefore, this model would be an ideal model for analysing human cerebral ischemia and the subsequent response.

## 3 APPLICATIONS OF BRAIN ORGANOIDS IN NEUROLOGICAL DEVELOPMENT AND DISEASE

Brain organoids can encapsulate key features of the human brain, including the distribution and organization of cells, physiological structures, point activities and neuronal networks. Although animal models of neurological disorders are crucial for studying the pathomechanisms of disease development, several unique human cognitive‐behavioural disorders, such as autism, microcephaly and other polygenic genetic disorders, are difficult to reproduce in animals.^84^, ^85^ Therefore, the use of induced pluripotent stem cells derived from these patients to prepare brain organoids has become a new tool to study the pathogenesis of these types of specific diseases (Table 2). The current disease models established using pluripotent stem cell‐derived brain organoids are primarily based on the diseases mentioned below.

**TABLE 2:** Application of brain organoids in nervous system development and disease.

Disease	Starting cell line	Genetic background	Features and applications of organoids	References
**Microcephaly‐related phenotypes**				
Primary microcephaly Zika virus infection‐induced microcephaly Seckel syndrome Aicardi–Goutières syndrome (AGS)	Microcephaly patient‐derived hiPSCs Microcephaly patient‐derived hiPSCs iPSCs iPSCs Seckel syndrome patient‐derived hiPSCs AGS patient‐derived hiPSCs	Carrying mutations in CDK5RAP2 gene. Carrying mutations in ASPM gene. Edited to delete WDR2 gene. Organoid infection with human Zika virus. Carrying mutations in CPAP gene. Carrying mutations in TREX1 gene.	A model of microcephaly to elucidate the mechanism of premature exhaustion of neural stem cells Microcephaly organoids, Elucidating the role of the ASPM gene in the development of microcephaly Smaller organoids, Premature neurogenesis and reduction in radial glia. Zika virus inhibits the ability of neural progenitor cells in organoids to proliferate, resulting in a decrease in organoid size. Novel drug screening of infected organoids for new treatments Elucidating the inhibitory effect of TREX1 mutants on organoids by astrocytes	Lancaster et al [55] LI et al [86] Zhang et al [87] Watanabe et al [88] Gabriel et al [89] Thomas et al [90]
**Macrocephaly‐related phenotypes**				
Macrocephaly Sandhoff disease	hPSCs Sandhoff disease patient‐derived hiPSCs	Gene‐edited for PTEN loss of function Carrying mutations in HEXB gene.	Macrocephaly organoids, Enhanced AKT signalling, transiently delayed neuronal differentiation. Accumulation of GM2 ganglioside, increased organoid size and higher progenitor proliferation.	Li et al [91] Allende et al [92]
**Autism spectrum disorder (ASD) related**				
ASD ASD with macrocephaly Rett syndrome	ASD proband‐derived hiPSCs ASD proband‐derived hiPSCs. Rett syndrome proband‐derived hiPSCs.	Cell‐of‐origin models, Misregulated expression of FOXG1 Cell‐of‐origin models Cell‐of‐origin models	ASD model, Misregulated expression of FOXG1 linked with higher production of GABAergic interneurons over glutamatergic excitatory neurons. Perturbed morphology of deep layer neurons Neural rosette phenotype with deregulated neurogenesis and miRNA expression.	Mariani et al [93] Schafer et al [94] Mellios et al [95]
**Other neurodevelopmental disorders**				
Miller Dieker syndrome (MDS) Down's syndrome Down's syndrome Fragile X Syndrome (FXS)	MDS patient‐derived hiPSCs hiPSCs hiPSCs iPSCs	Reduced organoid size and premature neural differentiation, Altered microtubule network organization in vRGC is abnormal Increase in GABAergic neurons larger size and more GFAP‐positive glial cells	Abnormalities in the N‐Cadherin/β‐Catenin pathway Up‐regulation of OLIG2 expression Decreased NPC proliferation, reduced neuronal cell differentiation and reduced proportion of glutamatergic neurons FMR1 gene affects neural progenitor cell proliferation	Iefremova et al [96] Xu [97] Tang [98] Brighi [99]

### 3.1 Microcephaly

Microcephaly is a condition in which the brain develops abnormally during embryonic development or during the first year of life, resulting in a reduction in brain size, with a high risk of mental retardation and seizures.^100^ Microcephaly includes primary microcephaly, and microcephaly induced by viral infections during pregnancy (e.g., Zika virus‐induced microcephaly) or caused by other syndromes (Seckel syndrome).

Primary microcephaly, also known as true microcephaly or autosomal recessive microcephaly, is largely caused by autosomal recessive mutations in genes that regulate centrosomes and assembly of cilia. However, the characteristics of rodent models of primary microcephaly are not the same as those observed in humans; for example, the rodent models do not show a significant reduction in brain size as that observed in humans.^101^ Therefore, it is crucial to establish a primary microcephaly organoid related to the human brain. In this regard, congenital microcephaly brain organoids carrying mutations in CDK5RAP2,^102^ CPAP,^89^ ASPM^86^ and WDR62^87^ have been established.

Reprogramming somatic cells obtained from patients with heterozygous truncated proteins encoded by the mutated CDK5RAP2 gene into induced pluripotent stem cells to generate brain organoids through a specific process revealed that, after neural induction, the cells generated less neuroepithelial tissue than controls.^55^ Moreover, the brain organoids had fewer radial glial cells (RGs) and more neurons, suggesting that deletion of the CDK5RAP2 gene leads to fewer progenitor cells and earlier neural differentiation. It has been shown that centrosome P4.1‐related proteins play a role in centriole formation and provide a scaffold for protein complexes during centrosome assembly.^103^ Compared to control organoids, brain organoids built using induced pluripotent stem cells derived from patients with Seckel syndrome (with mutations of the CPAP gene) resulted in an increase in the number and length of cilia and a delay in cilia disassembly in the organoids, thereby causing a delay in the cell cycle and resulting in earlier neuronal differentiation and smaller brain organoids size.^89^ WDR62 mutation‐ induced pluripotent stem cells generate brain organoids with delayed cilia disassembly, elongated cilia, and delayed cell cycle, leading to reduced proliferation and premature differentiation of neural progenitor cells.^87^ Whole‐exome sequencing identified microcephaly‐associated mutations in the NARS1 gene in more than 5000 patients with neurodevelopmental disorders, and patient‐derived induced pluripotent stem cells were used to establish cortical brain organoids with mutations of the NARS1 gene, which had small‐sized radial glial cells with reduced proliferation and cell cycle defects.^104^

ZIKV has been shown to infect the developing fetal brain and cause microcephaly. Due to the hazardous nature of infected human fetal tissues and variability of tissues obtained after post‐mortem, brain organoids have been widely used to mimic ZIKV infections and to study the underlying mechanisms.^105^ For example, forebrain organoids derived from ZIKV‐exposed hiPSCs revealed a specific tropism of ZIKV towards neural precursor cells, inhibiting neural precursor cell proliferation and increasing apoptosis, leading to a reduction in brain organoids size.^106^ Infected cells provide materials and mechanisms for viral production, leading to amplification of ZIKV and spread of infected cells over time. Transient exposure of early forebrain organoids to ZIKV is sufficient to cause the development of a microcephalic phenotype, including thinning of the neuronal layer, reduction in overall size, and dilatation of the ventricular cavities, which coincides with clinical signs of ZIKV infection in early pregnancy.^107^ Krenn et al.^108^ used ZIKV and HSV‐1 to infect human brain organoids to simulate the phenotype of microcephaly and found that the viral infection attenuated type I interferon immune response system in the organoids, which could not be observed in the conventional 2D culture system, demonstrating the superiority of 3D brain organoids in simulating neurodevelopmental diseases.

### 3.2 Miller Dieker syndrome

Miller‐Dieker syndrome (MDS) is the most severe form of classic anencephaly and is characterized by reduced brain volume, craniofacial malformations, mental retardation and seizures. Somatic cells obtained from patients with MDS were reprogrammed into hiPSCs and induced to be developed into brain organoids, which were significantly smaller than normal brain organoids at day 18.^109^ Increased apoptosis in the VZ‐like region, decreased vertical division, reduced neuroepithelial stem cell expansion and defects in mitosis of radio‐active extracellular neuroglial cells affected neocortical expansion, which led to the onset of MDS.^96^ Pathological changes associated with MDS can also be investigated using forebrain organoids specific for MDS patients that exhibit a significant reduction in the size of brain organoids and a shift from symmetrical to asymmetrical division of radial glial cells in the VZ. Altered organization of the microtubule network, disruption of cortical ecological niche structure, and altered expression of cell adhesion molecules, And this process was found to be regulated by the N‐cadherin/β‐catenin pathway, thus confirming the important role of the Wnt signalling pathway in the development of MDS through brain organoids.^96^ Thus, the study on MDS brain organoids has deepened the understanding of the pathogenesis of MDS and suggests that brain organoids have a wide range of applications in modelling human neurodevelopmental disorders.

### 3.3 Autism spectrum disorder

Autism spectrum disorder (ASD) is a developmental disorder of the brain characterized by language impairment, difficulties in social interaction and repetitive stereotyped behaviours.^110^ Mariani et al.^93^ induced iPSCs from autistic patients with macrosomia phenotypes to convert into telencephalic organoids and discovered that the neural progenitor cell cycle was shortened in the early stages of organoid development. An increase in the number of GABAergic neurons led to an excitatory/inhibitory neural imbalance caused by abnormalities in the FOXG1 gene and its downstream molecules. Wang et al.^111^ constructed iPSCs with a heterozygous deletion of the CHD8 gene using CRISPR‐Cas9 to form brain organoids. By comparing the transcriptomic data of brain organoids with deletion of the CDH8 gene and homozygous control, they found that the CHD8 gene regulates ASD‐related genes such as TCF4 and AUTS2, and these differential genes are also involved in neurogenesis, WNT/β‐catenin signalling pathway, and GABA neuronal differentiation. Qian et al.^112^ constructed a sliced neocortical organoid (SNO) that can circumvent the problem of cell death within the organoid due to lack of oxygen and nutrients in long‐term culture, thus generating a larger progenitor cell area and neural layer. Using this model, it was found that mutations in the autism susceptibility gene DISC1 resulted in impaired WNT/β‐catenin signalling and disrupted cortical neuron fate differentiation, which in turn led to abnormalities in the laminar markers SATB2, TBR1, ROB and CITP2. However, correcting the mutation of this gene using gene editing methods rescued these defects. Mutations in the RAB39B gene result in phenotypes such as large head malformations, autism and other abnormal phenotypes.

Zhang et al.^113^ found that brain organoids that carried the mutated RAB39B gene increased in size, and overproliferation of neural progenitor cells led to thickening of the SOX2^+^ VZ, which further led to differentiation disorders. Mechanistically, deletion of this gene caused overactivation of the PI3K‐AKT–mTOR signalling pathway, and inhibition of this pathway rescued the autism phenotype. Exploring the pathological mechanisms of ASD using brain organoids instead of the 2D system revealed changes in the cortical structure during development.

### 3.4 Down's syndrome

Down's syndrome, also known as trisomy 21, is a developmental disorder characterized by mental retardation caused by an abnormality in chromosome 21. Xu et al.^97^ found that overexpression of the oligodendrocyte transcription factor 2(OLIG2) gene on chromosome 21 led to overproduction of calretinin(CR)‐positive and somatostatin(SST)‐positive GABA neurons by culturing brain organoids from patients with Down's syndrome. Recently, Tang et al.^98^ performed single‐cell sequencing of brain organoids from patients with Down's syndrome cultured in vitro for 30d and 70d, respectively. They found delays in development of neural progenitor cells and neurons in the brain organoids. Immunostaining revealed a decreased proliferative capacity of neural progenitor cells, a significant decrease in deep and superficial cortical neuronal cells, and a decreased proportion of glutamatergic neurons in the brain organoids. Further studies revealed abnormalities in the DSCAM signalling pathway, and knockdown of the DSCAM gene or inhibition of the PAK1 signalling pathway using CRISPR/CAS9 rescued the phenotype related to cortical developmental defects in Down's syndrome.

### 3.5 Fragile X syndrome

Fragile X syndrome (FXS) is a mental retardation syndrome caused by mutations in the FMRP gene present on the X chromosome.^114^ Using CRISPR/Cas9, Brighi et al.^99^ constructed FMR1 gene‐truncated iPSCs and employed these to mimic some of the phenotypes of FXS in vitro using 2D neural cultures and 3D brain organoids. FXS brain organoids exhibited larger sized and more GFAP‐positive glial cells than homozygous control brain organoids, which is consistent with the observation that the FMR1 gene affects neural progenitor cell proliferation and glial cell differentiation under 2D culture conditions. These data suggest that this brain organoid model can be used to explore pathological mechanisms of FXS, thereby providing a new platform for treatment of such disorders.

### 3.6 Other neurodevelopmental disorders

However, current brain organoids are not capable of forming complex sulcal gyrus structure. Bershteyn et al.^109^ and Iefremova et al.^96^ used brain organoids to simulate the features of anencephalic gyrus malformation disease and discovered that brain organoids generated from patients with Miller‐Dieker syndrome have abnormal divisions of RG cells. Blair et al.^115^ found an increased proportion of glial cells with impaired neural differentiation in the TSC2 gene‐knockout 3D cortical spheroids and that addition of rapamycin rescued the phenotype of aberrant neural differentiation. This has important implications for exploring the pathogenesis and therapeutic approaches to the genetic disorder, tuberous sclerosis complex (TSC). Mellios et al.^95^ constructed brain organoids from a patient with Rett syndrome and found that mutations in the MECP2 gene caused abnormal proliferation and differentiation of NPCs. Birey et al.^43^ induced cortical and ventral cortical neurospheres and fused the two to form a brain organoid structure that mimicked the aberrant migration of GABAergic neurons in Timothy syndrome.

In summary, the use of human brain organoids to simulate early developmental features of neurodevelopmental diseases, such as neurogenesis, neuronal migration, cerebral cortical structure and functional networks, effectively compensates for the inadequacies of neuronal studies using 2D culture. In addition, some diseases that are difficult to study using mice or other animals as models, such as microcephaly and anencephalic gyrus malformation, can be reproduced more accurately with the help of brain organoids to reproduce abnormalities in neural stem cell proliferation and differentiation. Therefore, brain organoids are powerful tools for studying neurodevelopmental diseases.

## 4 APPLICATIONS OF BRAIN ORGANOIDS IN DRUGS SCREENING, AND TESTING

Brain organoids represent a promising avenue for the advancement of drug development, screening and testing procedures. The assessment of drug safety is a critical factor in the transition of pharmaceuticals to clinical phases. Owing to their substantial molecular and structural likeness to the original tissue, Brain organoids are likely to emerge as pivotal models in drug screening, offering the potential to mitigate the extensive testing required in clinical trials.

The application of brain organoids technology in ZIKV research has elucidated the correlation between ZIKV outbreaks and the incidence of congenital microcephaly. Moreover, it demonstrates significant potential in the field of drug testing, including the preparation of potential antiviral agents against the ZIKV.^116^ Brain organoids are also broadly applied in the screening of a range of other pharmaceuticals. Methamphetamine (METH) is recognized as an efficacious stimulant capable of inducing rapid states of euphoria. Nonetheless, it is frequently implicated in the onset of psychiatric disorders, depression and the degradation of the immune system.^117^ Xu et al.^118^ conducted a comprehensive screening of approximately 6000 compounds against ZIKV infection utilizing monolayer cultures of human neural precursor cells (hNPCs) derived from iPSCs and sophisticated brain organoids. The process of drug screening necessitates a homogenous cell population to manage the diversity in outcomes and to augment the reproducibility of the results.

Investigations utilizing brain organoids models, comprising neural progenitor cells, glial cells and neurons, have examined the effects of METH on the human brain. The research findings indicate that in brain organoids exposed to METH, there is an upregulation of specific cytokines, such as C‐X‐C motif chemokine ligand 8(CXCL8), and an increase in gene expression associated with neuroinflammation. This demonstrates the immune‐responsive capacity of brain organoids and suggests that METH treatment induces neuroinflammatory responses in these structures.^119^ Brain organoids technology introduces a novel approach to drug screening through the establishment of disease models, superseding conventional experimental models.

## 5 CURRENT LIMITATIONS AND FUTURE PERSPECTIVES OF BRAIN ORGANOIDS

Current research on pluripotent stem cell‐derived 3D brain organoid technology has achieved many milestones in addition to constructing models of brain development and neurological disorders. It has also been used to study cell division and human cortical progenitor cell expansion in human radial glia and for drug screening using brain organoids for prenatal drug treatment studies. Prenatal cocaine exposure in rats induces cytoarchitectural and related signalling changes in the embryonic neocortex, and differences in species render the use of simulated animal models alone insufficiently reliable.^120^ Lee et al.^121^ used a human brain organoid model to study the effects of prenatal drug exposure on the human fetal brain. Cocaine exposure inhibits the proliferation of neocortical progenitor cells, causes premature neuronal differentiation and interrupts the development of neural tissue, suggesting that cytochromeP450 3A5 (CYP3A5) may be a possible therapeutic target that can be used for the treatment of neurological developmental disorders. This suggests that brain organoids can help uncover good therapeutic targets and provide a new platform for research on perinatal drugs. Mansour et al.^79^ established a method to transplant human brain organoids into adult mouse brain, thus providing a functional in vivo model of brain organoid vascularization. They combined this method with optogenetic techniques to reveal functional synaptic connections between the transplanted brain organoids and the host, which provides an important reference for the establishment of brain development and neurological disease models under physiological conditions.

Despite the progress in pluripotent stem cell‐derived 3D brain organoids as mentioned above, some problems still remain to be solved. Brain organoids are not human brains in the true sense and are not able to self‐organize themselves into the exact shape and functional partitions as those of human brains.^122^ Moreover, brain maturation, especially at later stages, is highly dependent on vascularization of the SVZ, and the absence of blood vessel formation limits the supply of oxygen and nutrients to the organoids, often resulting in necrosis of the central region and interference with neuronal migration. Although brain organoids and neuronal cultures can produce functional synaptic connections, they cannot yet establish complete neural circuits with mature synapses.

It is expected that researchers will make significant advances in the study of improved organoid culture modes. As mentioned earlier, Ming's team^123^ developed the Spin Ω micro‐bioreactor, which facilitates the mass amplification and automated production of brain‐region specific organoids. Kirwan et al.^124^ successfully constructed an organoid model of the cortical neural network using a human iPSC line, which mimicked the development and functions of the cortical network and laid the foundation for simulation of complex psychiatric disorders. Pham et al.^125^ cultured iPSCs from patients to create vascularized brain organoids that more precisely mimic the brain anatomy and physiology in vivo, thereby improving hypoxia and nutritional support for brain organoids, which can facilitate brain disease modelling and also provide an ideal platform for drug testing. Schwartz et al.^126^ combined human iPSC‐derived neural progenitor cells, mesenchymal stem cells(MSCs), endothelial cells and microglial cell precursors on chemically engineered hydrogels to form 3D neural cultures that contained microglia and vascular networks. With the efforts of scientists in diverse fields such as neuroscience, stem cell biology, neurology, bioengineering and biomaterials, the traits and functions of brain organoids will more accurately replicate those of the human brain and will more precisely mimic human brain development and neurological disorders.

## 6 CONCLUSION

This review discusses important achievements and historical processes of the birth and development of pluripotent stem cell‐derived 3D brain organoid technology, including breakthroughs in pluripotent stem cell technology and the emergence of organoid technology. 3D brain organoids can more accurately simulate the human brain in terms of spatial structure and can also simulate some phenotypes related to brain development and neurological diseases that are difficult to achieve in animal models. Therefore, classic studies of pluripotent stem cell‐derived 3D brain organoids simulating brain development and neurological diseases are discussed and evaluated.

In addition, we present other research applications of 3D brain organoids and recent breakthroughs related to this technology in neuroscience. Finally, we present the current problems of 3D brain organoids, as well as an outlook on possible future improvements in this technology. In conclusion, brain organoids cannot self‐organize into shapes or functional brain compartments identical to those of the human brain, and they oxygen supply and nutrient support are limited due to the absence of vascularization. However, with technological breakthroughs in various fields, the traits and functions of pluripotent stem cell‐derived human 3D brain organoids will more closely simulate those of the human brain and will more fully play a role in mimicking human brain development and neurological disorders. Studies on 3D brain organoids will gradually help transform basic research into clinical applications and may have a significant potential to be used in many fields such as basic research, drug screening, and clinical treatment.

## AUTHOR CONTRIBUTIONS

**Yirizhati Aili:** Conceptualization (equal); data curation (equal); formal analysis (equal); software (equal); writing – original draft (equal). **Nuersimanguli Maimaitiming:** Conceptualization (equal); data curation (equal); investigation (equal); validation (equal); writing – original draft (equal). **Zengliang Wang:** Formal analysis (equal); funding acquisition (equal); project administration (equal); supervision (equal); writing – review and editing (equal). **Yongxin Wang:** Investigation (equal); resources (equal); supervision (equal); writing – review and editing (equal).

## FUNDING INFORMATION

This work was Sponsored by Natural Science Foundation of Xinjiang Uygur Autonomous Region (2022D01D70), Natural Science Foundation of Xinjiang Uygur Autonomous Region (20231107414).All the figures in the manuscript were drawn in Figdraw. The manuscript has been reviewed and approved by all the authors. All the authors have read the journal's authorship agreement and policy and declared that there was no conflict of interest.

## CONFLICT OF INTEREST STATEMENT

The authors declare that they have no competing interests.

## Data Availability

Data sharing is not applicable to this article as no new data were created or analysed in this study.
